# CCR5 Decorated Rilpivirine Lipid Nanoparticles Build Myeloid Drug Depots Which Sustains Antiretroviral Activities

**DOI:** 10.21203/rs.3.rs-4433306/v1

**Published:** 2024-06-03

**Authors:** Howard (E) Gendelman, Milankumar Patel, Sudipta Panja, Lubaba A. Zaman, Pravin Yeapuri, Shaurav Bhattarai, Santhi Gorantla, Linda chang, Alonso Heredia, Piotr Walczak, Samuel Cohen, Bhavesh Kevadiya

**Affiliations:** University of Nebraska Medical Center; University of Nebraska Medical Center; University of Nebraska Medical Center; University of Nebraska Medical Center; University of Nebraska Medical Center; University of Nebraska Medical Center; University of Nebraska Medical Center; University oif Maryland School of Medicine; University of Maryland; University of Maryland School of Medicine; Department of Pathology and Microbiology, University of Nebraska Medical Center, Omaha, NE; University of Nebraska Medical Center

**Keywords:** HIV, antiretroviral therapy, lipid nanoparticles, CCR5-targeting, non-invasive imaging

## Abstract

Antiretroviral therapy (ART) improves the quality of life for those living with the human immunodeficiency virus type one (HIV-1). However, poor compliance reduces ART effectiveness and leads to immune compromise, viral mutations, and disease co-morbidities. A novel drug formulation is made whereby a lipid nanoparticle (LNP) carrying rilpivirine (RPV) is decorated with the C-C chemokine receptor type 5 (CCR5). This facilitates myeloid drug depot deposition. Particle delivery to viral reservoirs is tracked by positron emission tomography. The CCR5-mediated RPV LNP cell uptake and retention reduce HIV-1 replication in human monocyte-derived macrophages and infected humanized mice. Focused ultrasound allows the decorated LNP to penetrate the blood-brain barrier and reach brain myeloid cells. These findings offer a role for CCR5-targeted therapeutics in antiretroviral delivery to optimize HIV suppression.

## Introduction

The human immunodeficiency virus type (HIV) global epidemic began in 1981 and has led to 40 million deaths and equal numbers of infected people.^1–3^ While HIV-1 replication suppressed by antiretroviral therapy (ART) has markedly improved disease outcomes, infection persists. Viral DNA integration into the cell target genome defines microbial latency, demonstrating that HIV-1 can circumvent antiretroviral immunity with continuous HIV comorbidities.^4–6^ ART faces challenges due to its failure to eliminate infection, the need for strict regimen adherence, and drug-related toxicities.^7,8^. ART bioavailability to the lymphoid, gut, central nervous system (CNS), heart, liver, and kidney tissues is added to this list of drug challenges.^4,9,10^ Limitations in ART tissue penetrance are linked to drug pharmacologic properties or ART pauses, where both lead to the emergence of viral drug resistance.^11^ Therefore, strict adherence to lifelong ART is required to maintain viral suppression.^12^

A means to improve these antiretroviral therapeutic challenges is long-acting (LA) ART. Improved drug delivery has an underexplored potential for improved therapeutic outcomes. One way this can be further improved is through functional lipid nanoparticles (LNPs). However, this alone may not allow drug accumulation to viral reservoirs.^13^ Success is made by optimizing antiretroviral drug (ARV) tissue and cell-specific targeting. LNPs may improve ARV pharmacodynamics by increasing drug circulation time and bioavailability.^4,12,14^ LNP composition and decoration enable ARV cell depots and enhance delivery to viral reservoirs.^15^ If a targeted system is realized, it can improve ARV’s residence time by creating a cell depot that could sustain antiretroviral activities.

Considering this idea, we created a C-C chemokine receptor type 5 (CCR5) decorated rilpivirine (RPV) LNP nanoprobe. The probe was designed to encase RPV with tissue delivery monitored by positron emission tomography (PET). The created LNP-RPV-CCR5 formulation led to viral suppression in viral reservoir tissues and cells in HIV-1_ADA_-infected hu-mice. ARV myeloid-targeted formulations produced cell depots and improved ARV antiretroviral responses.

## Results and discussion

### Synthesis and characterization of ^64^CuInEuS_2_ nanoprobes

**Figure 1:**
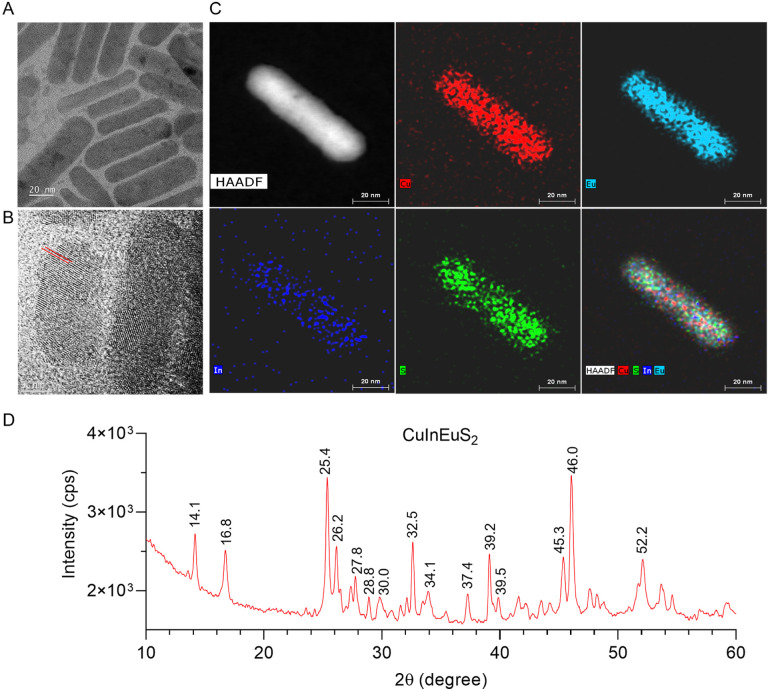
Morphological characterization of the multimodal CuInEuS_2_ nanoprobe. (A-B) Transmission electron microscope images of the multimodal rode shape nanoprobes. CuInEuS_2_ and its crystal lattice (red lines) are illustrated. (C) A scanning transmission electron microscopy map shows elemental localization within the nanoprobe from a corresponding high-angle annular dark-field electron microscopy image. The map shows the presence of copper (red), indium (blue), europium (cyan), and sulfur (green) in the nanoprobe. (D) XRD pattern of CuInEuS_2_ nanoprobe demonstrates a crystal structure.

Myeloid cell-targeted LNP imaging nanoprobe permits the simultaneous delivery and therapeutic antiretroviral activities. This theranostic system was created with RPV. The radioactive LNP-based imaging monitors drug delivery. To this end, a CCR5 targeting peptide was conjugated to PEGylated lipid (DSPE-PEG) by an acid amine coupling reaction combined with phosphatidylcholine (PC) and a peptide unconjugated DSPE-PEG to formulate the targeted LNP. RPV, an FDA-approved, nonnucleoside reverse transcriptase inhibitor) was co-encapsulated within the LNP, creating the LNP-RPV-CCR5. The nanoprobe’s properties and biodistribution were examined in humanized mice (hu-mice). The beta emitter^64^CuInEuS_2_ offered an ideal radioactive half-life due to its compatibility with PET computed tomography (CT) or magnetic resonance imaging (MRI) tracking. This tri-modality was selected based on known safety and ease in biological monitoring.^12,13^ The chelator-free ^64^CuInEuS_2_ nanoprobe was synthesized by solvothermal methods using thioacetamide, europium (III) chloride hexahydrate, indium (III) chloride dihydrate, and copper (II) chloride dihydrate at optimal molar ratios. The high boiling solvent, oleylamine, was selected for solvothermal synthesis to avoid narrowly dispersed particles. The nanoprobe morphology was examined under transmission electron microscopy (TEM) which revealed a narrowly dispersed rod-shaped nanostructure with an average diameter of 15 nm and an average length of 65 nm (Fig. 1A). High-resolution TEM images showed a crystal lattice (Fig. 1B). The presence of crystalllinity was confirmed by X-ray diffraction (XRD) peaks at 2θ of 14.1°, 16.8°, 25.4°, 26.2°, 27.8°, 28.0°, 30.0°, 32.5°, 34.1°, 37.4°, 39.2°, 39.5°, 45.3°, 46.0° and 52.2°, and electron diffraction patterns (Fig. 1D and Fig. S1A). High-angle annular dark-field scanning TEM showed copper (red), indium (blue), europium (cyan), and sulfur (green) coexistence within the ^64^CuInEuS_2_ nanoprobe (Fig. 1C). The results were supported by the appearance of the elements trace in energy-dispersive X-ray spectroscopy (Fig. S1B). ^64^CuInEuS_2_ was subjected to X-ray photoelectron spectroscopy (XPS) to evaluate its surface elemental composition (Fig. S2). The appearance of binding energy peaks corresponding to Eu 3d (at 1134.26 and 1164.40 eV), Cu 2p (at 931.87 and 951.69 eV), In 3d (at 444.07 and 451.58 eV), and S 2p (at 161.09 and 168.47 eV) demonstrated their rod-shape.

### Synthesis of a CCR5-peptide conjugated DSPE-PEG formulation

To formulate the CCR5-receptor-targeted LNP, a linear CCR5-peptide, D-Ala-Ser-Thr-Thr-Thr-Asn-Tyr-Thr-NH_2_, was selected as the targeting ligand. The free -NH_2_ group on the CCR5-peptide was conjugated to DSPE-PEG-NHS by an activated acid amine coupling reaction (Scheme 1). The ^1^H NMR spectra of the DSPE-PEG conjugated peptide showed chemical shifts at 0.8 and 1.27 ppm. These were assigned to the methyl and methylene protons of DSPE (Fig. S3). The chemical shift at 3.63 ppm was assigned to the methylene proton of PEG. The chemical shifts at 6.79 and 7.09 ppm were assigned to the 4-hydroxyphenyl ring protons of the tyrosine (Tyr), and those between 1.5 to 3 ppm and 4 to 4.5 ppm were transferred to the overlapping proton signals from the peptide and DSPE-PEG segments. ^1^H NMR confirmed both the CCR5-peptide and DSPE-PEG segments, affirming the synthesis of the DSPE-PEG-CCR5. High-resolution mass spectrometry results further supported its synthesis (Fig. S4).

### LNP physicochemical properties

**Figure 2:**
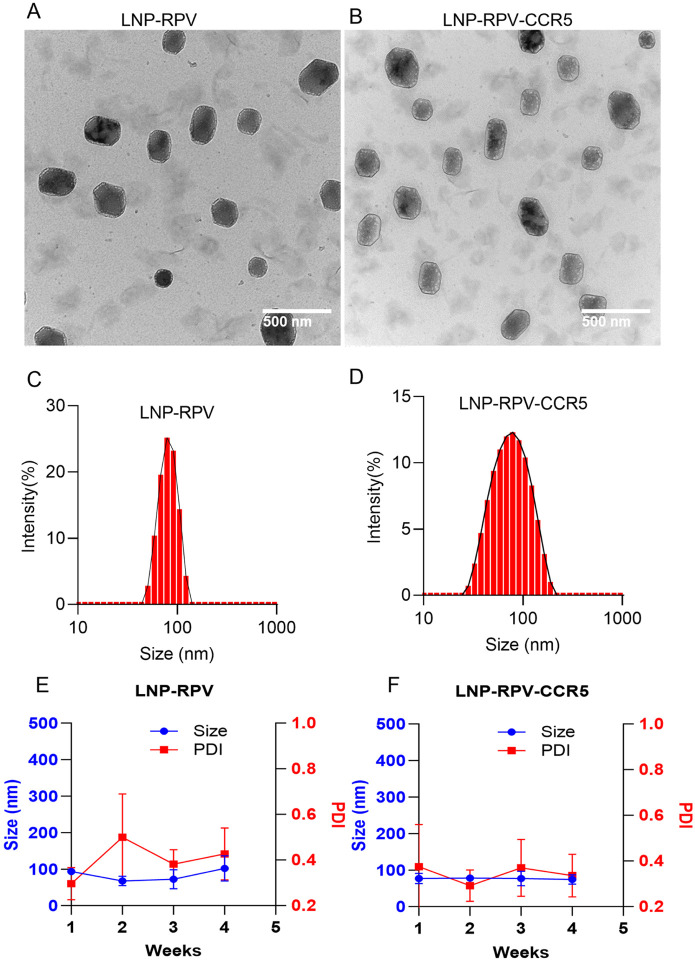
Physicochemical LNP characterization. (A-B) The TEM image shows a spherical LNP-RPV and LNP-RPV-CCR5 morphology. (C-D) The DLS size profile demonstrates the unimodular distribution of the LNPs. (E-F) The changes in size and polydispersity of the LNPs were recorded for one month at 4 °C. LNPs were stable without changes in size and polydispersity.

LNPs were prepared by microfluidic techniques by rapid, chaotic mixing of lipid and aqueous phases (Scheme 1B). The LNP-RPV-CCR5 contained a mixture of PC (45 wt%), DSPE-PEG (19.5 wt%), RPV (35 wt%), and DSPE-PEG-CCR5 (0.5 wt%) in its lipid phase. PBS was used in the aqueous phase (Table 1 and Scheme 1C). LNP without DSPE-PEG-CCR5, LNP-RPV was prepared in parallel to serve as a control formulation. To track the LNPs by PET, both LNPs were reformulated with 1.5 wt% of the radioactive nanoprobe ^64^CuInEuS_2_. TEM images showed a spherical morphology of both the LNPs (Fig. 2A–B). The particle sizes of LNP-RPV and LNP-RPV-CCR5 were 98 and 91 nm, respectively (Table 1 and Fig. 2C–D). LNPs were neutral in charge with a surface zeta potential from 0.29 to 0.37 mV (Table 1). LNPs with particle sizes of ~100 nm and a neutral surface charge were prepared for administration. The RPV loading content in LNP-RPV and LNP-RPV-CCR5 were 49.78 and 30.80 wt%, respectively (Table 1). The storage stability of the LNPs at 4 °C. LNPs showed no changes in particle size and dispersity for up to one month. These data demonstrated long-time storage stability (Fig. 2E–F).

### LNP treatment of human monocyte-derived macrophages (MDMs)

**Figure 3:**
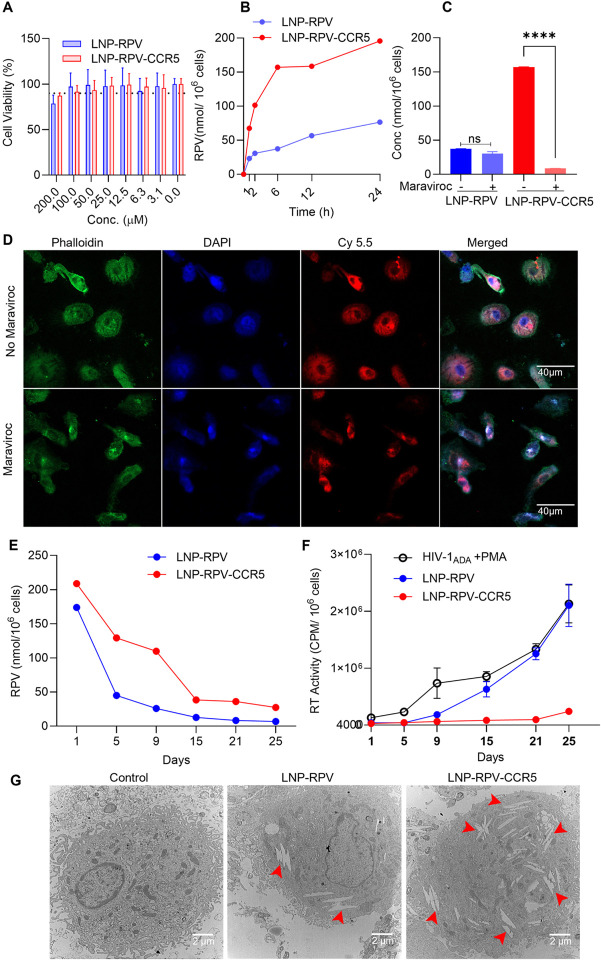
CCR5-receptor-modified LNPs facilitate the particle’s cell uptake, depot formation, and HIV-1 suppression. (A) Dose-associated macrophage viability measurements following LNP exposures by the CTB assay after a 24 h incubation. The LNP- RPV and RPV-CCR5 treatments showed that cell viability was maintained at 100 μM of RPV doses. (B) LNP-RPV and LNP-RPV-CCR5 macrophage uptake was analyzed by measuring RPV concentration for 24 h at 20 μM RPV. Comparisons between LNP-RPV and RPV-CCR5 showed a 3-fold increase in drug uptake. (C) The CCR5 inhibitor (maraviroc, 1 nM) attenuated LNP uptake for the LNP-RPV CCR5 LNP. (D) Confocal microscopy was performed with Cy 5.5 dye-labeled LNP-RPV-CCR5 treated macrophages in the presence (lower panel) and absence (upper panel) of maraviroc. (E) RPV retention and (F) viral suppression in LNP- RPV and RPV-CCR5 macrophage uptake were evaluated by measuring RPV and virus levels in the cell supernatant fluids. The data were collected over 25 days after a 100 μM administered dose. (G) TEM image of LNP engulfed macrophage. Macrophages depots for LNP-RPV-CCR5 are shown. Statistical analysis was performed by an unpaired t-test. ****, P < 0.0001; and ns=not significant.

Macrophages express CD4 and CCR5 receptors and are susceptible to HIV-1 infection.^14–16^ The cells are a known HIV reservoir.^17^ Infected macrophages can transmit the virus from person to person, serving as a depot for ARVs. Therefore, MDMs served as a primary cell model to examine the LNP antiretroviral efficacy. Before LNP treatment, the viability of MDMs was evaluated after 200 to 3 μM RPV LNPS exposures by the CellTiter-Blue Assay (Fig. 3A). The tests revealed a > 90% MDM viability with a dose equivalent of up to 100 μM RPV. To evaluate LNP uptake in MDMs 20 μM RPV was tested. The LNPs showed a time-dependent increase in RPV concentration for up to 12 h and slowly reaching equilibrium at 12 to 24 h (Fig. 3B). LNP-RPV-CCR5 showed a 3-fold higher RPV uptake at 12 h compared to equivalent LNP-RPV levels. To test whether cell uptake was CCR5 mediated, comparisons were made with and without the CCR5 antagonist, maraviroc (MVC). With 1 nM/10^6^ cell exposures MVC reduced LNP-RPV-CCR5 uptake. However, no MVC affect was seen for LNP-RPV (Fig. 3C). To affirm these results, Cy 5.5 dye-labeled LNPs with equivalent Cy 5.5 content were incubated with MDMs with or without MVC. After 4 h, MDMs’ nuclei and cell membranes were stained with DAPI (blue, stained DNA) and phalloidin (green, stained F-actin). The microscopic images revealed a bright red fluorescence (Cy 5.5) of LNP-RPV-CCR5 throughout the MDMs (Fig. 3D). However, differential LNP localizations with reduced fluorescence intensity was observed for MVC treatments. These data affirmed that the entry of LNP-RPV-CCR5 was blocked by inhibition of the CCR5-receptor. Moreover, MVC did not influence LNP-RPV uptake (Fig. S6).

These data affirm that the LNP-RPV-CCR5 cell entry principally followed a CCR5-receptor-mediated pathway. In addition, MVC treatment did not affect cytotoxicities as it revealed > 90% cell viability at 1 nM treatment dose (Fig. S5). The change in cell morphology in MVC-pretreated MDMs was due to the interaction between MVC and the CCR5 cell surface receptor.

To evaluate the RPV retention and viral suppression of the LNPs, phorbol 12-myristate 13-acetate (PMA) cell stimulation was used to maximize cell differentiation. The fully differentiated PMA-treated cells were then infected with HIV-1_ADA_ at the multiplicity of infection (MOI) 0.1 and treated with LNP-RPV or LNP-RPV-CCR5 at 100 μM RPV doses. After 24 h, treatment was removed, and cells were cultured in fresh media. Infected MDMs without LNPs were maintained as controls (HIV-1_ADA_ and PMA). On days 1, 5, 9, 15, 21, and 25, culture supernatant fluids were removed and then analyzed for HIV-1 reverse transcriptase (RT) activity. Cells were harvested in parallel to quantify RPV. On day 9, LNP-RPV showed 26 and 5 nmol RPV at 100 and 30 μM treatment doses (Fig. 3E and Fig. S7A). In contrast, LNP-RPV-CCR5 showed 109 and 50 nmol RPV at 100 and 30 μM treatment doses. Both LNPs demonstrated a dose-dependent RPV retention. Each showed higher RPV retention at 100 than 30 μM (Fig. 3E and Fig. S7A). In addition, the RPV retention followed descending trends over time throughout the treatment groups. At both doses, LNP-RPV-CCR5 demonstrated higher RPV retention than LNP-RPV. These results support the role of the CCR5 receptor in LNP-RPV-CCR5 cell uptake and its influence on the formation of the macrophage drug depot. To confirm these, LNP-containing macrophages were examined under TEM. Macrophages showed considerable RPV depots (red arrowhead, Fig. 3G) in LNP-RPV-CCR5 treated cells than for LNP-RPV. In parallel tests, HIV-1 RT activity assessed virion production in HIV-1_ADA_ infected control groups (Fig. 3F). A single 100 μM dose of LNP-RPV-CCR5 inhibited virion production for up to 25 days. In contrast, the LNP-RPV showed viral breakthrough after day 9 (Fig. 3F). The reduced efficacy of LNP-RPV in inhibiting HIV-1 replication was coordinated to lower RPV retention (5.10 nmol at day 9). The 100 μM dose of LNP-RPV-CCR5 restricted viral growth up to 25 days (Fig. S7B). At 30 μM of LNP-RPV-CCR5, the decreased RPV retention (3 nmol at day 25) was insufficient to inhibit viral growth (Fig. S7A).

### Biodistribution of LNPs in hu mice

**Figure 4:**
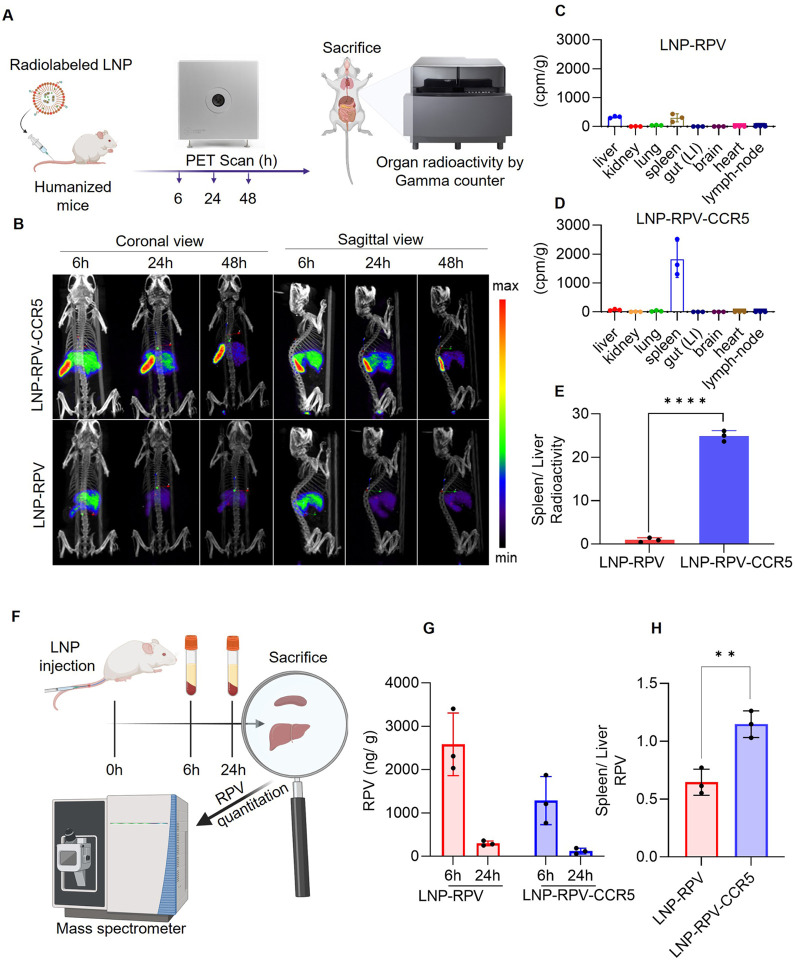
LNP and RPV tissue biodistribution in mice by PET imaging and mass spectrometry. (A) Schematic presentation of LNP biodistribution by PET. (B) Humanized mice were injected with LNP-RPV or RPV-CCR5, and particle biodistribution was monitored by PET at 6, 24, and 48 h. Both coronal (left panel) and sagittal (right panel) views for LNP-RPV-CCR5 are illustrated. (C-D) Quantitative measurements of radiolabeled LNPs in tissue were recorded at 48 h by gamma counter measurements. LNP-RPV-CCR5 was concentrated in the spleen. (E) Spleen/liver RPV ratios for LNP-RPV-CCR5 and LNP-RPV are shown. (F) A schematic presentation of LNP injection measurements in humanized mice is shown. (G) Plasma RPV levels following NP- RPV and RPV-CCR5 treated mice are illustrated at 6 and 24 h after injection. (H) The spleen/liver RPV ratio at 24 h shows the highest RPV levels in the spleen after LNP-RPV injection.

HIV uses CCR5 as a coreceptor for viral infection. The lack of this receptors in mice support their use *in vivo* studies of HIV infection in hu-mouse.^18^ Thus, hu-mice were used to evaluate the biodistribution of ^64^CuInEuS_2_ encapsulated theranostic LNPs. The biodistribution was performed with PET-CT bioimaging. Before bioimaging, we assessed the stability of the radiolabeled LNPs in mice plasma. These controls precluded any false positive signals. LNPs were incubated in 10% mice plasma at 37 °C to determine the radiolabeling stability. The total radioactivity in the LNP was measured in relation to the total radioactivity of the LNP-plasma solution. The radiolabeled LNPs were stable (98%, Fig. S8) in mice plasma after 24 h of incubation. This suggested their suitability for *in vivo* bioimaging. Radiolabeled LNPs (dose 1000 μCi/kg) were injected by tail vein to hu mice to assess particle biodistribution.^19^PET images were captured at 6, 24, and 48 h after injection and co-registered by CT (Fig. 4A–E). Both the coronal and sagittal PET-CT images demonstrated spleen and liver LNP distribution (Fig. 4B). The PET image displayed a progressive decrease of radioactive signals over time. This was attributed to the combined effect of radioactive decay and LNP excretion.^20^ Noticeably, LNP-RPV-CCR5 showed primary presence in the spleen, while LNP-RPV primarily accumulated in the liver. Comprehensively, the higher signal in LNP-RPV-CCR5 treated mice over LNP-RPV was linked to the tail vein injection site (Fig. S9). To validate these findings, mice were sacrificed at 48 h after injection, and the remaining radioactivity was assayed by a gamma counter (Fig. 4C–D). LNP-RPV-CCR5 showed a propensity to spleen tissue accumulation. In contrast, LNP-RPV was distributed throughout all examined tissues. LNP-RPV-CCR5 showed a substantially higher spleen/liver radioactivity ratio than LNP-RPV. The spleen harbors a significant number of CCR5-expressing immunocytes.^21^

To evaluate RPV distribution, LNP-RPV and LNP-RPV-CCR5 were injected at 25 mg/kg through the tail vein of the hu mice. The plasma RPV concentration was measured 6 and 24 h after injection (Fig. 4F–G). At 24 h, mice were sacrificed, and the liver and spleen RPV levels were determined by electrospray ionization mass spectrometry. At 24 h plasma, liver, and spleen RPV levels were 120, 4081, and 4600 ng/g in LNP-RPV-CCR5 treated mice. In contrast, RPV levels were 299, 2919, and 1898 ng/g in the LNP-RPV control mice. LNP-RPV-CCR5 demonstrated spleen-specific RPV accumulation with higher spleen/liver RPV ratios than for LNP-RPV (Fig. 4H). These data were well corroborated by PET imaging (Fig. 4B–E).

**Figure 5:**
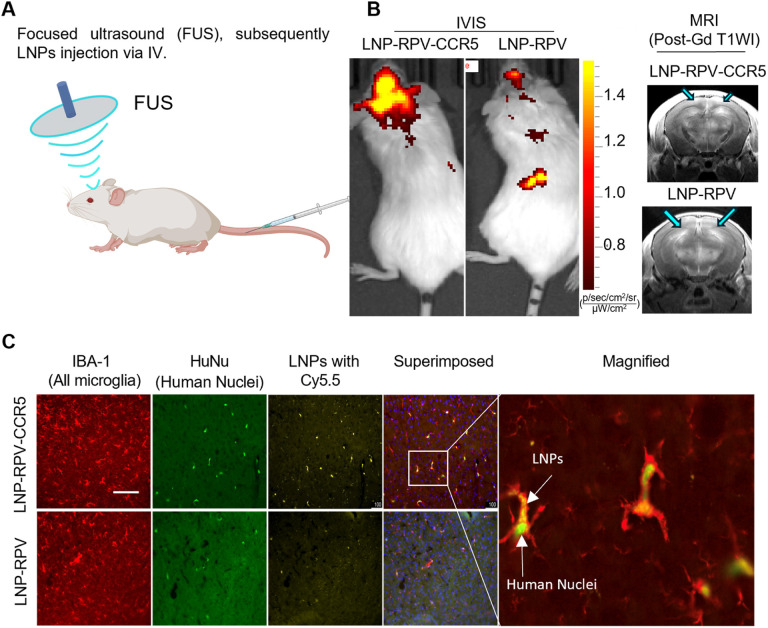
Brain delivery of LNP-RPV-CCR5 nanoparticles. (A) Schematic presentation of FUS-treated humanized microglial (MG) mice received LNP treatment. (B) IVIS images show bright yellow Cy5.5 signals in the brain of FUS-treated mice that received the LNP-RPV-CCR5 compared to LNP-RPV. Both mice showed good BBB disruption, as shown by the gadolinium enhancements (bright signals, blue arrows) on the coronal sections of the T1-weighted images (T1WI) on MRI. (C) Approximately 50% of microglia (IBA-1, red) showed human marker staining (HuNu, green), and only those showed the engulfment of LNPs. The Cy5.5 signals appear to be more intense in the animals that received the LNP-RPV-CCR5. Scale bar = 200 μm.

Yet another limitation of ARV biodistribution rests in penetrance to the brain viral sanctuary. Indeed, LNP-based cargo delivery rests in its limited penetrance across the blood-brain barrier (BBB) (Fig. S10). To affect the penetration of LNPs into the brain, we used focused ultrasound (FUS) combined with microbubble-induced BBB disruption (BBBd) in our hu mice (Fig. 5A). The verification of BBBd was affirmed by the gadolinium enhancements (bright signals, blue arrows). These changes were illustrated in the coronal sections of the brain T1-weighted MRI images (T1WI) (Fig. 5B). Immediately following FUS, mice were intravenously injected with Cy5.5 labeled LNPs. The FUS-mediated temporary BBB disruption allows the LNP to cross into the brain. After FUS, the BBB naturally reseals; a similar strategy can be applied to humans.^22^ On the following day, whole-body scans were performed with an *in vivo* imaging system (IVIS). This revealed a higher brain accumulation and retention of LNP-RPV-CCR5 than LNP-RPV (Fig. 5B). This data was affirmed by quantifying the elevated levels of RPV in brain tissue by mass spectrometry. Specifically, the brain tissue of the LNP-RPV-CCR5 treated hu mice showed RPV levels of 400 ng/g, compared to 55 ng/g in those treated with LNP-RPV. Although FUS facilitates the delivery of both LNPs to the brain, LNP-RPV-CCR5 showed the highest retention based on its interactions with CCR5 receptor-expressing human myeloid-microglial cells.^23^ To examine cell-specificity, brain tissue sections were stained with IBA-1 (red, microglia) and HuNu (green, human nuclei) and imaged by confocal microscopy (Fig. 5C). Approximately 50% of microglia (IBA-1, red) showed HuNu, green staining, and LNP engulfment. Mice treated with LNP-RPV-CCR5 displayed increased accumulation of LNPs in human microglia and higher cytoplasmic retention than those treated with LNP-RPV. These data support CCR5 targeted delivery.

### LNP-RPV-CCR5 viral suppression in hu-mice

**Figure 6:**
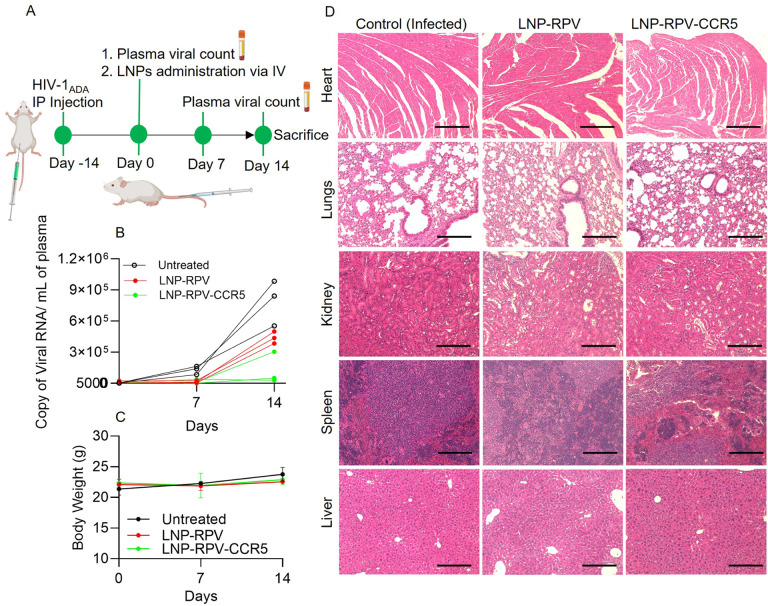
Viral suppression and toxicity profiles of LNP-carried RPV. (A) Schematic representations of experimental timelines are shown for LNP treatments. (B) Viral suppression efficiency of LNP-RPV (individual humanized mice) and LNP-RPV-CCR5 (averages of all animals) on days 7 and 14 are illustrated. LNP-RPV-CCR5 showed complete viral suppression up to day 14. (C) The change in mice body weight in the untreated, LNP-RPV, and LNP-RPV-CCR5 treated group. (D) The histological images of hematoxylin and eosin-stained heart tissues, lung, kidney, spleen, and liver sections from the treatment groups. The images were captured at 20X magnification.

After achieving higher levels of viral suppression in MDMs and lymphoid tissue-specific RPV biodistribution in human cell reconstituted hu-mice, an animal study was designed to validate levels of viral suppression for the LNP-RPV-CCR5. The timeline of the experiment is presented in Fig. 6A. Hu-mice were infected with 1.5 × 10^4^ tissue culture infectious dose 50 (TCID_50_) of HIV-1_ADA_. At two weeks, viral replication was confirmed by measuring plasma viral RNA copies. Subsequently, LNPs were administered by the tail vein injection at a dose of 25 mg/kg RPV equivalence. Viral suppression efficacy was analyzed by weekly plasma viral load measurements. Levels of viral suppression compared against LNP-RPV and LNP-RPV-CCR5 showed that the latter successfully held viral growth for 14 days in 2/3 treated mice (Fig. 6B). The higher levels of viral suppression of LNP-RPV-CCR5 were linked to CCR5-receptor-mediated lymphoid-specific RPV retention.

### Tissue toxicity measurements

To assess the potential LNP toxicity, the body weight of the hu-mice was measured. Blood samples were collected to determine hematologic profiles at the end of treatment. The heart, lung, spleen, liver, and kidneys were paraformaldehyde-fixed, sectioned, and stained with hematoxylin and eosin to assess tissue histology. The analysis of whole blood count and blood serum chemistry revealed no evidence of cytotoxicity in the LNP-treated group (Table S1 and S2). Moreover, the measured body weight remained unchanged throughout all the treatments (Fig. 6C). No histological abnormality was identified in the spleen despite the high levels of LNP accumulation and other examined organs (Fig. 6D). These limited examinations indicate that the LNPs were safe delivery vehicles. The results of these studies support the clinical translation of the LNP-based drug delivery.

## Conclusions

We successfully synthesized a multimodal radioactive nanoprobe with a CCR5-peptide conjugated DSPE-PEG-CCR5 lipid. The CCR5-targeted LNP-RPV-CCR5 and nontargeted LNP-RPV were formulated by microfluidic mixing. The spherically shaped LNPs had sizes near 100 nm with narrow size dispersity. These LNPs were devoid of associated toxicities at 100 μM RPV equivalence doses. Unlike LNP-RPV, LNP-RPV-CCR5 demonstrated substantially higher macrophage uptake and retention. In macrophages, the RPV was retained as a drug depot. A single dose of LNP-RPV-CCR5 treatment demonstrated a 25-day-long viral suppression in the HIV-1 infected macrophages not seen by LNP-RPV treatments. The CCR5-receptor-mediated RPV uptake and depot formation were linked to an extended viral suppression. The PET-CT theranostic imaging revealed a spleen-specific biodistribution of LNP-RPV-CCR5, resulting in a higher RPV accumulation in the spleen of hu mice, a key HIV reservoir. The FUS combined with microbubble-induced BBBd facilitated the delivery of LNPs to the brain and higher drug retention in human microglia. Moreover, a single dose of LNP-RPV-CCR5 was sufficient to hold viral growth at bay in HIV-1 infected hu mice. The therapeutic efficacy of the CCR5-targeted delivery system can be propelled by synergistic combination of multiple ARVs for longer-term effective HIV-1 treatments.

## Materials and Methods

### Materials

Copper (II) chloride dihydrate (307483), Indium (III) chloride (334065), Europium (III) chloride hexahydrate (203254), Thioacetamide (163678), Oleic acid (364525), Oleylamine (969831), 1-octadecene (O806–1L), L-α-phosphatidylcholine (PC) (from egg yolk), 3-(4,5-dimethylthiazol-2-yl)-2,5-diphenyltetrazolium bromide (MTT), were obtained from Sigma Aldrich, St. Louis, MO, USA. Phorbol 12-myristate 13-acetate (PMA; P8138), 1-octanol, paraformaldehyde (PFA), were obtained from Sigma Aldrich (St. Louis, MO, USA). Dulbecco’s modified eagle’s medium (DMEM) containing glucose (4.5 g/L), phosphate-buffered saline (PBS), gentamicin, L-glutamine, sodium pyruvate, AcroMetrix EDTA Plasma Dilution Matrix (S2284) were purchased from Thermo Fisher Scientific/Gibco (Waltham, MA, USA). Heat-inactivated pooled human serum was purchased from Innovative Biologics (Herndon, VA). Cell Titer BlueTM (CTB; G8080) was purchased from Promega (Madison, WI, USA). Rilpivirine (RPV; A904176) was purchased from Amadis Chemical (Zhejiang, China). 1,20-distearoyl-phosphatidylethanolaminemethyl-polyethyleneglycol conjugate 2000 (DSPE-PEG2000), DSPE-PEG_2000_ carboxy NHS and DSPE-PEG(2000)-N-Cy5.5 were purchased from Avanti Polar Lipids (Birmingham, AL, USA). The CCR5 targeting peptide, D-Ala-Ser-Thr-Thr-Thr-Asn-Tyr-Thr-NH2 was purchased from P3 BioSystems (Louisville, KA, USA). The radioactive 64 copper chloride (^64^CuCl_2_) was requested and delivered from the Washington University School of Medicine MIR cyclotron facility (St. Louis, MO, USA).

### Synthesis of radioactive nanoprobes

The multimodal nanoprobe particles were prepared by the solvothermal method. At first, the thioacetamide (15.026 mg, 2 mmol) and oleylamine (6 mL) were taken in a 20 mL glass vial, and the reaction mixture was sonicated for 2 min (operated at 20%, Cole-Parmer 750 W model CPX750, IL, USA). In a separate vial, indium (III) chloride dihydrate (22.118 mg, 1 mmol), europium (III) chloride hexahydrate (36.64 mg, 1 mmol), 1-octadecene (10 mL), and oleic acid (6 mL) were homogenized by vigorous stirring. The homogeneous solution was transferred to the reaction mixture and further probe sonicated for 5 min. Subsequently, the copper (II) chloride dihydrate (34.09 mg, 2 mmol) was also added to the reaction mixture. The reaction mixture was quickly transferred to the Teflon-lined hydrothermal autoclave and heated at 280°C for 8 h. After the autoclave cooled down to room temperature, the crude reaction mixture was dispersed in ethanol (50 mL) by sonication. The solution was then spun down (950 × *g* for 30 minutes at 20°C) and decanted off the supernatant. This ethanol-washing step was repeated thrice to remove unreacted starting materials. The particles were then stored in a desiccator for future use. To make the radioactive nanoprobe, the copper (II) chloride was substituted with radioactive copper (II)-64 chloride.

The bulk morphology and crystal lattice structure of the CuInEuS_2_ nanoprobe were characterized by high-resolution transmission electron microscopy and selected area electron diffraction, respectively. The elemental composition and chemical color mapping were analyzed by energy-dispersive X-ray spectroscopy and scanning transmission electron microscopy (STEM) with high-angle annular dark-field (HAADF) (FEI Tecnai Osiris S/TEM operated at 200 kV), respectively. The nanoprobe’s surface composition and crystal structure were analyzed by XPS (Thermo Fisher Scientific, Waltham, MA, USA) and powder XRD (Rigaku SmartLab Diffractometer, Rigaku Corporation, Tokyo, Japan). The thermal property was analyzed by differential scanning calorimetry (NETZSCH DSC 204 F1 Phoenix, Waldkraiburg, Bayern, Germany) and thermogravimetric analysis (NETZSCH TGA 209 F1 Libra system, Waldkraiburg, Bayern, Germany).

### Synthesis of CCR5 peptide conjugated DSPE-PEG-CCR5 formulations

A linear peptide with a sequence of D-Ala-Ser-Thr-Thr-Thr-Asn-Tyr-Thr-NH_2_ has been selected as a CCR5-receptor targeting ligand. The free amine group of the peptide was conjugated with PEG lipid, DSPE-PEG_2000_ carboxy NHS via an activated acid-amine coupling reaction. The reaction was conducted in a Schlenk tube by dissolving the peptide (3.57 mg, 4.15 μmol) in anhydrous DMSO (250 μL) followed by the addition of DIPA (10 μL). After 30 min of reaction at room temperature, DMSO dissolved DSPE-PEG_2000_ carboxy NHS (10 mg, 3.47 μmol) was added to the reaction mixture and allowed to stir for the next 24 h. The completion of the reaction was examined by thin-layer chromatography using 1:5 (v/v) MeOH/DCM as eluent. After the completion, the crude reaction mixture was dialyzed (cellulose acetate, MWCO 3.5kDa) against deionized water (DI) to remove the unconjugated peptide. The lyophilization of the dialyzed product ensued in a floppy white solid, further characterized by ^1^H NMR spectroscopy.

### LNPs formulation and their physicochemical properties

LNPs were formulated by rapidly mixing the lipid and aqueous phases in the microfluidic device. To formulate CCR5-targeted and RPV-encapsulated LNPs (LNP-RPV-CCR5), L-α-phosphatidylcholine (PC, 45 wt%), DSPE-PEG2000 (17.5 wt%), RPV (37 wt%), and DSPE-PEG-CCR5 (0.5 wt%) were combined in lipid phase and performed the microfluidization (Precision Nanosystem) with PBS as an aqueous phase (Table 1). The LNP without CCR5 ligand (LNP-RPV) was also formulated and used as a control in various experiments. The existing lipid phase was combined with 1.5 wt% of ^64^CuInEuS_2_ to formulate radiolabel LNP and 0.5 wt% of DSPE PEG(2000)-N-Cy5.5 lipid to formulate Cy5.5-dye-labeled LNPs. During LNP formulation, the ratio of the aqueous phase to the lipid phase was maintained at 3:1 (v/v), and the total flow rate was held at 12 mL/min. After microfluidization, the LNPs were purified by dialyzing (3.5–5 kDa cut off, cellulose acetate) against DI water over two days. The purified LNP was further passed through a 40 μm cell strainer to remove the unencapsulated drug precipitate. The size and zeta potential of LNP were measured by DLS (Zetasizer Nano ZS, Malvern). The long-term stability of LNP at 4 °C was evaluated by intermittently measuring their size and zeta potential over a month. The radioactivity of nanoprobe ^64^CuInEuS_2_ was measured by gamma-ray scintillation spectrometry. (Hidex AMG). To assess the RPV content, the LNPs (50 μL) were sonicated with methanol (250 μL) for 30 min and subjected to ultra-high performing liquid chromatography (UPLC, Acquity UPLC H-class^®^ system, Waters Milford, MA, USA). Correspondingly, the Cy5.5-lipid content in the LNP was calculated by measuring the fluorescence (Ex/Em = 683/703 nm) using a benchtop plate reader (Molecular Devices, SpectraMax M3, Sunnyvale, CA). The bulk morphology of the LNPs was captured under the transmission electron microscope (TEM, FEI TECNAI G2 Spirit TWIN microscope, USA). To determine the drug loading content (LC), a known volume of LNP solution was lyophilized and the total mass content (drug + lipid) per mL of LNP was evaluated. The LC was determined by following the equation, (RPV per mL × 100)/ total mass of the LNP per mL.

### Plasma stability of the radiolabeled LNP

The stability of the radiolabeled LNPs was determined by incubating them in 10% mice plasma at 37 °C for 24 h. After the incubation, the LNP-plasma solution’s total radioactivity was determined by gamma counter. To measure the radioactivity outside the LNP, the LNP-plasma solution was filtered by using centrifugal filtration (Amicon^(R)^, 10K molecular-weight cut-off) at 2,000 × g for 15 min and measured the radioactivity in the filtrate. The percent of radiolabeling stability was as follows, radiolabeling stability (%) = [(total radioactivity) - (radioactivity in the filtrate)] × 100/total radioactivity.

### Cell cultures

Monocytes were obtained by leukapheresis from HIV and hepatitis B seronegative donors^24^. Monocytes were cultured in 10% human serum (heat-inactivated) supplemented Dulbecco’s modified Eagle medium (DMEM) containing glucose (4.5 g/L), L-glutamine (200 mM), sodium pyruvate (1 mM), gentamicin (50 μg/mL), ciprofloxacin (10 μg/mL) and recombinant human macrophage colony-stimulating factor (1,000 U/mL) at 37°C in 5% CO_2_ incubator. On every other day, half of the culture media was replaced with fresh media and continued for one week to facilitate MDMs. The MDMs were then incubated with PMA (50 ng/mL) containing media for 24 h and used for ex vivo assays.

### Cell viability assay

The effect of LNPs on MDMs cell viability was evaluated by Cell Titer BlueTM (CTB) assay. MDMs containing 96 well plates (1.5 × 10^5^ cells/well) were incubated with LNPs at a dose ranging from 3 to 200 μM equivalent to RPV for 24 h. After the allotted time, the cells were further incubated with CTB solution (20 pL/well) at 37°C for 2 h, and fluorescence (E_x_/E_m_ = 560/590 nm) intensity was recorded on bench top plate reader (Molecular Devices SpectraMax M3, SoftMax Pro 6.2 software). The percentage of cell viability in the treatment group was evaluated by comparing their fluorescence intensity with that of the untreated group. Analogously, the MDM cell viability against MVC was evaluated at a dose ranging from 0.5 to 4 nM.

### LNP MDM uptake

To evaluate the uptake, LNPs at doses equivalent to 30 and 100 μM RPV were incubated with MDMs in a 12-well plate (1.0 × 10^6^ cells/well) with and without the pretreatment of MVC (1 nM). The uptake of LNP was determined by the means of RPV uptake. The concentration of RPV in MDMs was measured at 1, 2, 6, 12, and 24 h of post incubation. At each time point, MDMs were washed and scraped into PBS. The scraped cells were pelleted down by centrifugation and sonicated with HPLC grade methanol (200 μL) to extract the RPV. To remove the cell debris, the methanol solution was further centrifugated (at 5000×g for 10 min), and the supernatant was used to measure RPV concentration by UPLC.

### Antiretroviral activity and LNP RPV macrophage retention

HIV-1 RT activity was employed to determine the antiretroviral efficacy. MDMs were challenged with HIV-1_ADA_ (1.5 × 10^4^ TCID50/mL) at 0.1 MOI for 8 h. The cells were then washed with PBS and cultured overnight in fresh media. On the following day, HIV-1 infected cells were treated with LNPs at the dosage of 30 and 100 μM RPV equivalent for 24 h. The treatment was then removed by PBS wash, and the cells were cultured in fresh media. At 1, 5, 9, 15, 21, and 25 days of post-treatment removal, culture media were collected to analyze the RT activity and the associated cells were harvested to quantitate RPV retention.

### Animals

NSG (NOD.Cg-Prkdc^scid^ Il2rgt^m1Wjl^/SzJ) mice were obtained from the Jackson Laboratories, Bar Harbor, ME, and bred under specific pathogen-free conditions at the University of Nebraska Medical Center by the ethical guidelines set forth by the National Institutes of Health for the care of laboratory animals. The mice were humanized (hu-mice) by following the previously published protocol.^25^ Humanization was confirmed by flow cytometry analysis of blood immune cells (CD45 and CD3) staining.

### LNP biodistribution

PET imaging was performed on hu-mice to assess the real-time biodistribution of radio-labeled (^64^CuInEuS_2_) LNPs. The LNPs were injected to hu-mice at the dosage equivalent to 1000 μCi/kg by the tail vein. The biodistribution of radio-labeled LNPs was acquired at 6, 24, and 48 h of post-injection using the PET bioimaging system (MOLECUBE β-CUBE, NV, Ghent, Belgium). The co-registration of 3D computed tomography (CT) and PET was performed by using VivoQuant 3.5 software (Invicro Boston, MA, USA). At 48 h post-injection, mice were sacrificed, and major organs were collected, weighed, and measured the radioactivity using gamma scintillation spectrometry (Hidex Automatic Gamma Counter, Turku, Finland). The radioactivity count percent was determined by following the equation:

$$\text{Radioactivitycount(\%/g)}=\frac{\left(\text{organradioactivitycount}\;\times \;100\right)}{\left(\text{totalradioactivitycount}\;\times \;\text{organweight}\right)}$$

To determine the biodistribution of RPV, the nonradioactive LNPs were injected at the dose of 25 mg/kg equivalent to RPV via the tail vein. At 24 h of post-injection, mice were sacrificed, and liver, spleen, and blood plasma samples were collected for RPV quantification by electrospray ionization mass spectrometry (Waters ACQUITY H-class UPLC, Xevo TQ-S micro-mass spectrometer, MA, USA)

To conduct the brain distribution study, hu mice were anesthetized and prepared for the focused ultrasound (FUS) procedure. This involved removing the scalp hair and inserting a 26-gauge intravenous catheter into the tail vein. After stereotaxic localization of the bregma, 100uL of Definity^®^ microbubble solution (1:1000 dilution by volume) was immediately injected before the FUS. The FUS with optimized parameters (500kHz frequency, 1.0W power, 10% duty cycle, and 75s duration) was then applied to each mouse hemisphere (+/− 2.5 mm of bregma). After the FUS, LNPs were immediately infused slowly through the tail vein catheter. The animal was then taken to the 9.4 Tesla MR scanner (Biospec Avance III Bruker MR scanner) for verification of the blood-brain barrier disruption (BBBd) with T1-weighted MRI before and after intravenous gadolinium infusion (25% dilution). On the following day, the IVIS (Xenogen Corporation, Alameda, CA ) was performed to assess the brain distribution of LNPs and compare those with or without FUS. Following perfusion and euthanasia, we performed immunofluorescence evaluations on the brain tissue. This included staining for all nuclei (DAPI), microglia (IBA-1), and human nuclei (HuNu).

### HIV-1 suppression in hu-mice

Hu-mice with an average age between 18 to 20 weeks were infected with 1.5 × 10^4^ tissue culture infective dose_50_ (TCID50) of HIV-1_ADA_ via intraperitoneal injection. At 2 weeks post-infection, blood samples were collected via submandibular vein bleeding, blood plasma was 10-fold diluted in AcroMetrix^™^ EDTA plasma dilution matrix (Catalog # S2284, Thermo Scientific, USA) and subjected to viral load determination using automated COBAS Ampliprep V2.0/Taqman-48 system (Roche Molecular Diagnostics, Basel, Switzerland). After confirmation of plasma viral load, mice were separated into three groups: HIV-1 infected, untreated control, LNP-RPV, and LNP-RPV-CCR5. LNPs were injected via tail vein at the dose of 25 mg/kg RPV equivalent. The body weight and plasma viral load were determined on days 0 and 7 and 14 of post-LNP injection. On day 14 of post-LNP injection, the mice were terminated, and blood and major organs collected. Blood samples were used for whole blood cell count (by Abaxis VetScan HM5) and serum chemistry (by Abaxis VetScan VS2), and the tissues from the major organs were fixed, paraffin-emedded, and stained with hematoxylin and eosin (H&E). The histological images of different tissues were captured on a Nuance EX multispectral imaging system affixed to a Nikon Eclipse E800 microscope.

### Statistical analysis

Data are presented as mean ± standard deviation. The statistical difference between the two groups was analyzed using an unpaired t-test. The p < 0.05 was considered statistically significant. All statistical analyses were performed using GraphPad Prism 10.2.2.397 (GraphPad Software, Inc., San Diego, CA).
